# Clinical Characteristics Associated with Antibiotic Treatment Failure for Tuboovarian Abscesses

**DOI:** 10.1155/2016/5120293

**Published:** 2016-02-17

**Authors:** Huma Farid, Trevin C. Lau, Anatte E. Karmon, Aaron K. Styer

**Affiliations:** ^1^Department of Obstetrics and Gynecology, Beth Israel Deaconess Medical Center, Boston, MA 02215, USA; ^2^Department of Obstetrics, Gynecology and Reproductive Biology, Harvard Medical School, Boston, MA 02115, USA; ^3^Vincent Department of Obstetrics and Gynecology, Massachusetts General Hospital, Boston, MA 02114, USA; ^4^Vincent Reproductive Medicine and IVF, Massachusetts General Hospital, Boston, MA 02114, USA

## Abstract

*Objective*. Although parenteral antibiotic treatment is a standard approach for tuboovarian abscesses, a significant proportion of patients fail therapy and require interventional radiology (IR) guided drainage. The objective of this study is to assess if specific clinical factors are associated with antibiotic treatment failure.* Study Design*. Retrospective medical record review of patients hospitalized for tuboovarian abscesses from 2001 through 2012 was performed. Clinical characteristics were compared for patients who underwent successful parenteral antibiotic treatment, failed antibiotic treatment necessitating subsequent IR drainage, initial drainage with concurrent antibiotics, and surgery.* Results*. One hundred thirteen patients admitted for inpatient treatment were identified. Sixty-one (54%) patients were treated with antibiotics alone. Within this group, 24.6% failed antibiotic treatment and required drainage. Mean white blood cell count (K/*μ*L) (18.7 ± 5.94 versus 13.9 ± 5.12) (*p* = 0.003), mean maximum diameter of tuboovarian abscess (cm) (6.8 ± 2.9 versus 5.2 ± 2.0) (*p* = 0.03), and length of stay (days) (9.47 ± 7.43 versus 4.59 ± 2.4) (*p* = 0.002) were significantly greater for patients who failed antibiotic treatment.* Conclusions*. Admission white blood cell count greater than 16 K/*μ*L and abscess size greater than 5.18 cm are associated with antibiotic treatment failure. These factors may provide guidance for initial selection of IR guided drainage.

## 1. Introduction

Tuboovarian abscesses (TOAs) are a common complication of pelvic inflammatory disease (PID) and affect approximately 10–15% of women with PID [1]. Previously, it was assumed that* Chlamydia trachomatis* and* Neisseria gonorrhea* infections were the primary causative agents of TOA [2]. However, it is now known that this disease process is often polymicrobial. In most cases, TOAs represent a significant complication of an ascending lower genital tract infection which evolves to PID and involves the uterus and adnexal structures. TOAs may also be caused by inflammatory bowel disease, appendicitis, or diverticulitis, with direct local spread of bacteria to the fallopian tubes from the gastrointestinal tract [3]. Following upper genital tract involvement, a significant inflammatory response ensues, resulting in local tissue necrosis, proliferation of anaerobic organisms, and abscess formation within the fallopian tube and ovary [4].

Historically, the treatment of TOAs involved a total abdominal hysterectomy with bilateral salpingo-oophorectomy, resulting in significant morbidity and infertility [4]. With the advent of antibiotics targeting anaerobes and gram negative aerobes, broad-spectrum intravenous (IV) antibiotics became the first-line treatment for TOAs, with treatment success ranging from 75% to 85% in some case series [1, 5]. However, due to the variable rates of success with antibiotic therapy alone, minimally invasive drainage techniques have been developed and utilized over the past three decades either as a secondary treatment option or as the initial treatment approach.

Percutaneous drainage of abdominal abscesses of all etiologies was described by Johnson et al. in 1981, after the group had been performing percutaneous drainage guided by either CT or ultrasound since 1976 for abscesses, with a success rate of 89% [6]. In 1986, Worthen and Gunning applied this technique to TOAs and reported success rates ranging from 77% to 94% [7]. Subsequent studies confirmed success rates for transvaginal ultrasound-guided drainage of TOAs ranging from 78% to 100% [2, 8–10]. Levenson et al. reported a success rate of 95% with CT-guided or ultrasound-guided drainage of 57 TOAs in 49 patients. These investigators found that TOAs of gynecologic etiology were more likely to be treated successfully with interventional radiology (IR) guided drainage than those caused by appendicitis, Crohn's disease, diverticulitis, or other gastrointestinal diseases [10].

Despite the success rates of IR abscess drainage and the inconsistent success rate of exclusive parenteral antibiotic treatment, the Centers for Disease Control and Prevention (CDC) recommends antibiotic therapy as the first-line treatment for TOAs [11]. Although a proportion of patients will fail parenteral antibiotic treatment for TOA and require abscess drainage, there are no standardized guidelines to direct clinicians as to when either antibiotics or IR drainage treatment is the most appropriate initial option. It is ideal to expand our understanding of pretreatment risk factors for antibiotic treatment failure and the subsequent clinical course following treatment failure. To this end, the objectives of this study were to assess if specific clinical characteristics are associated with antibiotic treatment failure and to investigate how treatment failure impacts outcome (length of stay).

## 2. Methods

This study was approved by the Partners Healthcare Institutional Review Board (IRB number 2012-P-001483/1). A retrospective electronic medical record review of women admitted for the inpatient care of TOA at the Massachusetts General Hospital (MGH) from January 1, 2001, through December 31, 2012, was performed. Potential subjects were identified with the use of ICD-9 codes for PID (614.9), acute salpingitis and oophoritis (614.0, 614.3), acute pelvic peritonitis (614.5), and TOA (614.2). A broad range of ICD-9 codes were used in order to capture all patients who might have been admitted with a TOA. One thousand ninety-three electronic and hardcopy charts were reviewed to confirm eligibility. Inclusion criteria included the following: confirmation of TOA by imaging and clinical criteria according to CDC criteria (abdominal or pelvic pain and one or more of the following: cervical motion tenderness, uterine tenderness, or adnexal tenderness [12]) and inpatient admission greater than 24 hours to our institution for treatment. Women who declined admission or did not have a TOA confirmed by clinical criteria and imaging were excluded.

Patient records were reviewed for demographics, medical and surgical history, and treatment undertaken for TOA. All subjects underwent radiographic imaging, which included combined real-time pelvic and abdominal ultrasound, computed topography (CT), or magnetic resonance imaging (MRI). The majority of subjects had multiple imaging modalities performed prior to final diagnosis per clinician discretion. The largest dimension (cm) of the TOA from the radiology reports was used. Subjects were categorized into four groups based upon treatment course: IV antibiotic treatment only (MED), failed IV antibiotic treatment requiring subsequent interventional radiology drainage (IRD) during the initial hospitalization, initial IR drainage with concurrent IV antibiotics (MED/IRD), and initial surgical intervention (SURG). Management of patients was decided upon by the evaluating clinical team, and the decision to treat patients medically or surgically was based on clinical status and the discretion of the medical team. There is currently no standard of care, which is what prompted the study.

The primary dependent variable was the rate of treatment failure, and the primary independent variables were clinical characteristics associated with treatment failure. Treatment failure was defined as persistent pain and/or fever following initial IV antibiotics for 48–72 hours necessitating IR drainage. The secondary dependent variable was mean length of stay (days) among respective treatment groups. Patients who were successfully managed with IV antibiotics (MED) were designated as the reference treatment group to which the other groups were compared. Continuous and categorical variables were analyzed within treatment groups. Chi-square tests were performed for categorical variables, and *t* test analyses were performed for continuous variables with the use of EpiCalc 2000 (version 1.02, Brixton Health, Llanidloes, UK, 1998), with *p*-values < 0.05 designating statistical significance. Demographic, clinical, and reproductive characteristics were compared for patients among all treatment groups. Descriptive variables were expressed as means and proportions and analyzed with univariate analyses. A multivariate logistic regression model was fit to evaluate the independent relationships between failed antibiotic therapy and age, maximum TOA dimension, and white blood cell count (WBC) at the time of admission (SAS version 9.3, SAS institute, Inc., Cary, North Carolina).

## 3. Results

Of the 1,093 charts reviewed, 113 patients met inclusion criteria, and the remainder did not have a TOA by imaging criteria. Sixty-one (54%) patients initially underwent treatment with antibiotics alone. Within this group, 24.6% failed treatment with antibiotics alone and required IR drainage (IRD). Demographic characteristics are provided in Table 1 and were similar across all groups. The mean age for the entire study population at the time of admission was 40.4 ± 13.1 years of age (yo) (range: 16 to 75 yo). Thirty-six percent of patients were nulliparous, and 63% were Caucasian. Six percent were HIV positive, and 6% had multiple concurrent partners.

Admission clinical characteristics were analyzed for the entire cohort. Subjects reported a mean of 4.78 ± 4.62 days of abdominal/pelvic pain prior to presentation, although women in the SURG group had the fewest days of abdominal/pelvic pain prior to admission, with a mean of 2.6 ± 2.16 days of pain (*p* = 0.003). Forty-three percent of patients demonstrated cervical motion tenderness on pelvic exam. Upon presentation, the mean oral temperature was 100.2 ± 1.97°F, and the mean white blood cell count was 15.4 ± 5.38 K/*μ*L. Upon admission, 73% were tested for gonorrhea and chlamydia; none were positive for* N. gonorrhoeae* and 7% tested positive for* C. trachomatis.* The majority of patients (112/113) underwent an imaging modality: 74% underwent transvaginal/abdominal ultrasounds, 75% underwent CT, and 6% underwent MRI. Sixty-five percent of subjects underwent two imaging modalities, most commonly pelvic ultrasound (PUS) and CT. Mean TOA size was 6.31 ± 3.0 cm. Twenty-six percent of patients had bilateral TOAs, and the mean dimension of the largest TOA in bilateral cases was 7.56 cm ± 3.62 cm. Forty-four percent were determined to have a gynecologic etiology of their TOAs, and the rest of the TOAs were due to a gastrointestinal etiology. The frequency of etiology was similar among all treatment groups, with the exception of appendicitis, which was found more commonly in the SURG group (44%, *p* = 0.006).

Symptoms upon presentation, frequency of unilateral abscess, and admission temperature were similar between patient groups. However, when these clinical characteristics were further analyzed by treatment group, there were significant differences in clinical characteristics, specifically white blood cell count and TOA size (Table 2).

Subjects in the IRD group had the greatest admission mean WBC (18.7 K/*μ*L ± 5.94 K/*μ*L) of the entire study population (*p* = 0.03). There was a statistically significant difference in mean WBC between the MED and IRD groups (13.9 K/*μ*L versus 18.7 K/*μ*L, *p* = 0.003) and between the MED/IRD and IRD groups (15.0 K/*μ*L versus 18.7 K/*μ*L, *p* = 0.026). Admission WBC greater than 16.0 K/*μ*L increased the odds of failing antibiotic treatment (odds ratio: 22.0; 95% CI 2.3–201.2, *p* trend: 0.006 (Table 3)).

Subjects in the SURG group also had the largest mean dimension of TOA (7.85 ± 3.96 cm, *p* = 0.001). Compared to the IRD group, subjects in the MED group had smaller mean maximum diameter of TOA (5.18 cm (SD 2.05) versus 6.78 cm (SD 2.95), *p* = 0.03). Logistic regression demonstrated that maximum dimension of TOA greater than 5.2 cm was predictive of antibiotic failure (odds ratio: 1.5; 95% CI 1.1–2.0, *p*-value = 0.0169). Age was not associated with the likelihood of antibiotic treatment failure (Table 3).

The mean inpatient length of stay (LOS) across the entire cohort of patients was 5.8 days ± 4.2 days. Compared to subjects in the MED group, women in IRD and SURG groups had the greatest inpatient LOS (9.47 days, *p* = 0.002, and 6.77 days, *p* = 0.006, resp.) as seen in Table 4. Subjects in the IRD group had the longest LOS of all women in the entire cohort. Compared to women who underwent initial IR drainage and concurrent IV antibiotics (MED/IRD), LOS in the IRD group was 4 days longer (4.85 days versus 9.47 days, *p* = 0.008). Women in the IRD group underwent IR drainage 2.5 days later than women in MED/IRD group (3.2 days versus 0.58 days, *p* = 0.001).

The four most common antibiotic regimens were as follows: gentamicin/clindamycin (14%), second generation cephalosporins/doxycycline/flagyl (11%), fluoroquinolone/flagyl (11%), and aminopenicillin/fluoroquinolone/flagyl (11%). The frequency of use of antibiotic regimens was similar among all the treatments (data not shown). Only one patient in the MED group and one patient in the MED/IRD group had positive blood cultures. Thirty-seven percent of patients had positive microbiology cultures. There was no statistically significant difference in the frequency of specific abscess organisms that were isolated among groups who either underwent IR drainage or had surgery. In the SURG group, 20% of patients' microbiology cultures were positive for* E. coli*, and 13% were positive for Gram Positive Cocci (GPC) along with* Peptostreptococcus*. In the MED/IRD group the microbiology data was notable for 27% of patients with the combination GPC, Gram Negative Rods (GNR), and Gram Positive Rods (GPR) and 18% with* E. coli*. In the IRD group, 38% of patients' microbiology data were positive for* E. coli* (although 40% of patients had other bacteria in addition to* E. coli* growing in the culture), and 23% were positive for GNR. An association between microbiology culture data and risk of treatment failure was not observed (data not shown).

## 4. Discussion

The current CDC clinical guidelines recommend medical management as the initial treatment approach for TOAs in women who are hemodynamically stable with abscess size less than 9 cm and no signs or symptoms of abscess rupture [12]. However, studies have demonstrated that antibiotics have maximum success rates of approximately 85% [1] and mean success rates approaching 70% [4]. Minimally invasive techniques of TOA drainage are an alternative to antibiotic treatment alone, and when compared directly with IV antibiotics alone, some investigators have demonstrated that drainage may be the more successful treatment. Despite the lower than expected success rates of medical treatment and the widespread availability of IR, it still remains unclear who may benefit most from exclusive medical treatment versus concurrent medical treatment and image-guided drainage as the initial treatment approach. This lack of consensus lies in a limited ability to identify patients who may be at greatest risk for antibiotic failure at the time of initial presentation. As a result, this study was conducted to assess if specific admission clinical parameters are associated with antibiotic treatment failure and to assess if outcomes are affected following failure of initial antibiotic treatment.

This study demonstrated that WBC greater than 16 K/*μ*L and TOA size greater than 5.18 cm were predictive of treatment failure when IV antibiotics were exclusively used as the initial treatment for TOA. Interestingly, subjects who failed antibiotic treatment and required IR drainage had the highest WBC count of the entire cohort, whereas subjects who were treated successfully with IV antibiotics had the lowest WBC count (Table 2). These findings suggest that white blood cell count may be a useful parameter to help guide treatment choice. However, there may be limitations to using leukocytosis as a marker of the severity of infection in cases with bacteremia or other inflammatory illnesses or comorbidities.

Worthen and Gunning recommend transvaginal ultrasound-guided drainage of TOAs in conjunction with antibiotics as the first-line treatment for TOAs of any size [7]. DeWitt et al. described that patients with a mean TOA size of ≤6.3 cm were successfully treated with medical management alone [13]. In contrast to that study, this analysis found that the upper limit of mean abscess size was 5.18 cm for successful antibiotic treatment, and the success of antibiotic therapy was not impacted by antibiotic regimen. We realize that there may be significant variation in the size of greatest dimension depending on the specific imaging modality. In this study, a majority of patients had either a pelvic CT or MRI to confirm initial ultrasound measurements, and this may have been contributory to variability in measuring TOA abscess dimensions. Interestingly, the percentage of patients with bilateral TOAs in the MED/IRD group and the percentage of patients with bilateral TOAs in IRD group were similar (Table 3). Based upon the findings of this study, concurrent antibiotic and abscess drainage may be considered as an ideal treatment for the case when TOA is greater than 5 cm. However, we appreciate that several factors such as patient comorbidities, patient or clinician preference, and the availability of interventional drainage resources may also impact the decision to proceed with concurrent drainage at the time of admission.

Greenstein et al. examined the treatment outcomes of 122 patients with TOAs, 65.6% of whom were treated successfully with antibiotics alone and 34.4% of whom failed antibiotic therapy and required operative intervention. The vast majority of operative interventions were surgical, and only 2 patients underwent IR drainage. They reported that increasing WBC count, TOA size, older age, and increasing parity were significant predictors of failure of antibiotic therapy. Mean TOA size treated successfully with antibiotics was 4.4 cm compared to 7.3 cm in the surgical group. Mean white blood cell counts for patients in the surgical group were 15.91 K/*μ*L compared to 12.36 K/*μ*L in the medical treatment group [14]. Our findings are consistent with this study, although we found that abscesses up to 5.18 cm could be treated successfully with antibiotics alone. A significant point of difference is that Greenstein et al.'s study did not include any patients who underwent IR drainage prior to antibiotic treatment and only 2 who underwent IR drainage after antibiotic failure. In contrast, our study included 41 patients who underwent IR drainage initially or after failed antibiotic treatment. As a result, the findings of our study may be more applicable to institutions with available resources and expertise with IR drainage. Since our study analyzed each treatment group separately, this study was able to assess the demographic and clinical differences between the patients who were treated successfully with IR drainage compared to those who failed antibiotic treatment. The results of our study demonstrated that the IRD group and MED/IRD group were similar in clinical characteristics. However, since the patients in the IRD group required IR drainage, it seems that this group of patients was incorrectly identified as lower risk when they were assigned to antibiotic therapy alone. In contrast to Greenstein et al., who recommended trial of IV antibiotics in all patients, the findings of our study support concurrent antibiotics and IR drainage as the initial treatment approach when TOA is greater than 5 cm.

Multiple studies have demonstrated excellent success rates for IR drainage. Perez-Medina et al. randomized 40 women with unilateral TOAs less than 10 cm in maximal diameter to IV antibiotics or IV antibiotics and transvaginal ultrasound-guided drainage. They discovered that 90% were treated successfully with drainage and antibiotics compared with 65% of patients with antibiotics alone [15]. Goharkhay et al. found similar results when comparing women managed with IV antibiotics alone or with drainage and antibiotics. In their study sample, all eight patients who underwent drainage and concomitant antibiotics had complete resolution of their symptoms, while 50% of patients who received only IV antibiotics failed treatment and required either drainage or surgery [16]. Gjelland et al. reported a similar success rate of 93.4% with transvaginal ultrasound-guided drainage of TOAs in 302 women who received concurrent IV antibiotics [17]. These studies support our findings that IR drainage may be considered a first-line treatment option in select patients.

Length of stay is of particular interest in this emerging era of cost-effective health care. This study observed an association of increased LOS with antibiotic treatment failure. Subjects in the IRD group had the longest LOS, whereas those in the MED and MED/IRD groups had the shortest lengths of stay (Table 4). Perez-Medina et al. reported that patients undergoing drainage were discharged 5 days sooner than patients treated with antibiotics alone [15]. However, our study only observed a greater LOS in patients who failed antibiotic therapy. The increase in length of stay may have resulted from the fact that treatment failure with antibiotics was not recognized until after several days of hospitalization, as patients in the IRD group did not undergo drainage for a mean of 3.2 days. This practice correlates with previous recommendations that patients be treated with at least 48 to 72 hours of antibiotic treatment prior to transitioning to an alternative treatment. These findings highlight the significance of this study's aim to identify characteristics of those who are at increased risk of antibiotic failure and who may benefit from concurrent abscess drainage and a potential reduction of overall morbidity and LOS. Ideally, if utilized in patients identified as high risk for antibiotic treatment failure, IR drainage may minimize the risk of an extended LOS and reduce excessive inpatient care costs.

There were several limitations to consider in this study. There were a limited number of patients who failed antibiotic therapy and ultimately underwent abscess drainage. While we were able to observe statistically significant differences in several clinical characteristics of patients and in the primary outcome (length of stay), the study was likely underpowered to demonstrate additional characteristics associated with antibiotic treatment failure. Additional limitations included the single institution retrospective cohort design of this study, which may limit the generalizability of the results. A notable strength of this study was the comparison of outcomes among several types of TOA management: medical, drainage with interventional radiology, and surgical ones. Since all aspects of patient care were rendered from a single institution, there was greater consistency in management decisions and similar technique and protocols in drainage during the study period. Although our study did not suggest that antibiotic failure corresponded with any specific antibiotic regimen, this would be of interest for future investigation to assess if a specific antibiotic regimen modifies the risk of medical treatment failure in specific subsets of patients.

A significant point to consider is that the clinical presentation for TOA can be variable, and it may be quite challenging to identify which patients may be at increased risk of antibiotic treatment failure. The decision regarding initial treatment for patients with TOAs may be impacted by several clinical factors during initial presentation and not by a limited number of discrete laboratory values at the time of admission. Interestingly, patients in the MED and IRD groups had similar demographic and admission clinical characteristics (Tables 1 and 2) with the notable exception of differences in WBC count and TOA size. Based upon our findings, the IRD group seems to have been incorrectly identified as low risk. Moreover, the use of admission clinical characteristics may serve to identify those subsets of patients at greater risk for antibiotic treatment failure.

In summary, this study provides additional insight into clinical characteristics which may identify patients who are at risk to fail conservative parenteral antibiotic therapy and who may benefit from concurrent abscess drainage with antibiotic treatment. In select patients, concurrent abscess drainage may represent the most optimal treatment option. Previous studies have demonstrated the efficacy and safety of TOA drainage, and the findings of this study support the feasibility of initial drainage of patients with specific thresholds of TOA size and leukocytosis. Additional larger observational studies will be necessary to provide further guidance and standardization of treatment for TOA.

## Figures and Tables

**Table 1:** Demographic characteristics by treatment group.

Demographics	MED (*N* = 46)	MED/IRD (*N* = 26)	IRD (*N* = 15)	SURG (*N* = 26)	All patients (*N* = 113)
Age (years) (mean ± SD)	38 (11.33)	37 (11.91)	45 (20)	46 (10.49)	40.4 (13.1)
Nulliparous	18 (41%)	18 (72%)	3 (23%)	9 (35%)	39 (36%)
Caucasian	18 (40%)	19 (73%)	9 (60%)	17 (74%)	79 (63%)
African American	8 (18%)	2 (8%)	2 (13%)	4 (17%)	16 (13%)
Hispanic	15 (33%)	5 (19%)	3 (20%)	0 (0%)	23 (18%)
Prior gonorrhea/chlamydia infection	8 (21%)	2 (9%)	1 (8%)	0 (0%)	11 (13%)
Current contraception use	17 (49%)	12 (57%)	3 (38%)	12 (63%)	66 (59%)
History of Bilateral Tubal Ligation (BTL)	0 (0%)	4 (19%)	1 (13%)	2 (11%)	16 (19%)

MED: intravenous antibiotic treatment only.

MED/IRD: initial interventional radiology drainage with concurrent intravenous antibiotics.

IRD: failed intravenous antibiotic treatment requiring subsequent interventional radiology drainage.

SURG: initial surgical intervention.

**Table 2:** Admission clinical characteristics by treatment group.

	MED^*∗*^ *N* = 46	MED/IRD *N* = 26 (*p*)	IRD *N* = 15 (*p*)	SURG *N* = 26 (*p*)
Duration of pain prior to presentation (days) (mean ± SD)	5.49 (4.87)	5.2 (5.68) *0.82*	4.25 (2.7) *0.4*	2.6 (2.16) ^*∗∗*^ *0.03*
Presence of cervical motion tenderness (CMT)	22 (58%)	8 (44%) *0.26*	1 (20%) *0.13*	0 (0%) ^*∗∗*^ *0.01*
Admission temperature (F) (mean ± SD)	100.6 (1.84)	99.7 (2.02) *0.08*	100 (2.03) *0.3*	99.9 (2.13) *0.2*
Mean white blood cell count (WBC), K/*μ*L (SD)	13.9 (5.12)	15 (3.97) *0.3*	18.7 (5.94) ^*∗∗*^ *0.003*	16.5 (5.82) ^*∗∗*^ *0.05*
Unilateral abscess	30 (71%)	19 (76%) *0.28*	12 (80%) *0.38*	20 (80%) *0.31*
Largest dimension of TOA (cm) (SD)	5.18 (2.05)	7.42 (3.22) ^*∗∗*^ *0.02*	6.78 (2.95) ^*∗∗*^ *0.03*	7.85 (3.96) ^*∗∗*^ *0.001*

^*∗*^ *MED is reference group for comparisons*.

^*∗∗*^ *p* < *0.05 denotes statistical significance*.

MED: intravenous antibiotic treatment only.

MED/IRD: initial interventional radiology drainage with concurrent intravenous antibiotics.

IRD: failed intravenous antibiotic treatment requiring subsequent interventional radiology drainage.

SURG: initial surgical intervention.

**Table 3:** Assessment of the likelihood of antibiotic treatment failure.

Variable	Odds ratio (95% confidence interval)	*p*-value
Age	1.1 (1.0–1.1)	0.0590
Maximum dimension of TOA	1.5 (1.1–2.0)	^*∗∗*^0.0169
WBC tertile		0.0049
WBC < 13	Reference group	—
WBC ≥ 13 and <16	8.0 (0.9–74.5)	0.0684
WBC ≥ 16	22.0 (2.4–201.2)	^*∗∗*^0.0063

^*∗∗*^denotes *p*-value < 0.05.

**Table 4:** Outcomes by treatment group.

	MED^*∗*^ (*N* = 46)	MED/IRD (*N* = 26) (*p*)	IRD (*N* = 15) (*p*)	SURG (*N* = 26) (*p*)
Length of stay (days) (mean ± SD)	4.59 (2.4)	4.85 (3.02) *0.6*	9.47 (7.43) ^*∗∗*^ *0.002*	6.77 (4.13) ^*∗∗*^ *0.006*
Duration of inpatient antibiotics (days) (mean ± SD)	4.47 (7.19)	3.54 (2.75) *0.53*	7.77 (6.21) *0.14*	5.71 (4.09) *0.51*
Duration of outpatient antibiotics (days) (mean ± SD)	13.7 (3.84)	13.3 (4.03) *0.79*	12.2 (2.8) *0.24*	11.2 (3.3) ^*∗∗*^ *0.04*

^*∗*^ *MED is reference group for comparisons*.

^*∗∗*^ *p* < *0.05 denotes statistical significance*.

MED: intravenous antibiotic treatment only.

MED/IRD: initial interventional radiology drainage with concurrent intravenous antibiotics.

IRD: failed intravenous antibiotic treatment requiring subsequent interventional radiology drainage.

SURG: initial surgical intervention.
